# Serum Cytokine Profile in Patients with Candidemia versus Bacteremia

**DOI:** 10.3390/pathogens10101349

**Published:** 2021-10-19

**Authors:** Saad J. Taj-Aldeen, Fayaz Ahmad Mir, Siveen K. Sivaraman, Atqah AbdulWahab

**Affiliations:** 1 Microbiology Division, Department of Laboratory Medicine and Pathology, Hamad Medical Corporation, Doha P.O. Box 3050, Qatar; 2 Department of Biology, College of Science, University of Babylon, Hilla P.O. Box 4, Iraq; 3 Qatar Metabolic Institute, Academic Health Systems, Doha P.O. Box 3050, Qatar; FMir1@hamad.qa (F.A.M.); ssivaraman@hamad.com (S.K.S.); 4 Department of Pediatrics, Sidra Medicine and Hamad Medical Corporation, Doha P.O. Box 3050, Qatar; atqah2015@gmail.com

**Keywords:** candidemia, bacteremia, risk factors, interleukins

## Abstract

Bloodstream *Candida* infections constitute a major threat for hospitalized patients in intensive care units and immunocompromised hosts. Certain serum cytokines play a decisive role in anti-microbial host defense. Cytokines may act as discriminatory biomarkers that can significantly increase in candidemia compared to bacteremia patients. The concentration of secreted cytokine/chemokines was determined using a multiplexed cytometric bead array run on a cell analyzer. The cytokines tested during the study were interleukin (IL)-1β, IL-6, IL-17A, IL-10, IFN-γ, IL-4, IL-2, IL-8, IL-12p70 and the tumor necrosis factor (TNF)-α. The cytokines of 51 candidemia patients were characterized and compared to the cytokine levels of 20 bacteremia patients. Levels were significantly elevated in patients with bloodstream infections compared to healthy controls. Cytokines comprising IL-2, IL-17A, IL-6 and IL-10 were significantly elevated in the patients with bloodstream *Candida* infection as compared to the patients having bloodstream bacterial infections. The levels were found to be promising as a potential diagnostic marker for bloodstream *Candida* infections.

## 1. Introduction

The incidence of candidemia has increased dramatically, including the infections documented in intensive care units (ICUs). For example, 53% of documented candidemia in Hamad hospital, Qatar, was from the ICUs [1]. *Candida* spp. are the third most common microorganisms responsible for health-care-related bloodstream infections [2]. However, blood cultures for yeasts lack sensitivity and need prolonged incubation (> 48 h) to generate positive results. As a consequence, antifungal drugs are often prescribed either prophylactically, preemptively, or empirically in high-risk patients [3]. The resulting overuse of antifungal drugs may lead to the emergence of *Candida* species that are resistant to azoles and/or echinocandins [4,5].

The early diagnosis of fungal infection has become increasingly important in order to prevent invasive candidiasis. There are some reports suggesting that C-reactive protein (CRP) and procalcitonin (PCT) can be used to diagnose bacterial sepsis [6]; however, their role and other cytokines in diagnosis of fungal infections has not been clearly demonstrated. Host immunity is of clear importance for controlling *Candida* infections. Currently employed clinical characteristics do not differentiate between fungal and bacterial infections. Interleukins, promptly and transiently produced in response to infections and tissue injuries, contribute to host defense through the stimulation of acute phase responses, hematopoiesis and immune reactions [7,8]. This retrospective study aims to assess the risk factors associated with candidemia in ICUs and patients at high risk, to measure the serum levels of inflammatory cytokines of Th-1, Th-2 and Th-17 lineage and to compare them with those observed in the cases of bacteremia. Though several studies have documented changes of cytokines and chemokines in bacteremia or sepsis, few studies have investigated candidemia or compared the differences between candidemia and bacteremia [9,10]. Therefore, in our study, we investigated the profiles of various cytokines that are involved in the regulation of systemic inflammation in high-risk patients. The cytokines investigated were interleukin (IL)-1β, IL-6, IL-17A, IL-10, IFN-γ, IL-4, IL-2, IL-8, IL-12p70 and the tumor necrosis factor (TNF)-α.

## 2. Materials and Methods

### 2.1. Study Design and Patients

This retrospective study was a single-center analysis from January 2016 to December 2018. The acquired sera were stored at −80 °C until analysis. We analyzed clinical information pertaining to bloodstream infections (BSIs) from 71 (51 candidemia and 20 bacteremia) patients hospitalized in all clinical departments of Hamad Medical Corporation (HMC), including the intensive care units (ICUs), hematology/oncology department and other medical and surgical wards. The study subject population was composed of all adult and pediatric hospitalized patients of both genders who developed candidemia or bacteremia. A *Candida* BSI was defined when one or more cultures of blood from patients with relevant clinical signs and symptoms were positive for a *Candida* species [11]. All patients selected for further analyses had at least one blood culture positive for a *Candida* species, as identified by the HMC Microbiology Laboratory database. Only the isolate from the first culture of blood collected from each patient at the time of onset of candidemia was included. The use of retrospective laboratory data and preserved blood sera within this study was reviewed and approved by the Medical Research Center (MRC) Ethics Committee at Hamad Medical Corporation (approval number 16149/16). The requirement for written informed consent was waived because of the blind retrospective and observational nature of this study.

### 2.2. Data Collection and Definitions

Demographic characteristics and underlying medical conditions were recorded systematically for each case. Clinical conditions and risk factors within minimum 10 days prior to candidemia were also recorded, including the presence of intravenous and total parenteral nutrition (TPN), mechanical ventilation and renal replacement therapy. We defined ICU population as patients hospitalized in ICUs at the time of candidemia and conversely for non-ICU population.

A total of 71 serum samples from patients from adult and pediatric wards of HMC were obtained, including 51 that yielded *Candida* spp. and 20 that yielded bacteria. Ten serum samples from healthy people without infection were used as control samples for comparison. Venous blood samples were collected in vacutainer tubes containing ethylene diamine tetraacetic acid (EDTA) under sterile conditions. Serum was obtained after centrifugation at 1300 rcf and immediately stored frozen at −80 °C until processed.

The definitions of nosocomial infections were established according to the definitions provided by the Center for Disease Control and Prevention (CDC). The mortality rates observed within the 30 days after the development of candidemia were calculated.

### 2.3. Isolation and Identification of Pathogens

Automated Bactec™ (Becton Dickinson, Sparks, MD, USA) blood culture systems were used during the study period. Yeasts and bacteria isolated from blood cultures were identified by MALDI-TOF mass spectrometry (Microflex Mass Spectrometer, Bruker Daltonics GmbH, Bremen, Germany) as described previously [1,12]

### 2.4. Measurement of the Serum Cytokine Levels

The concentration of secreted cytokine/chemokines was determined using a multiplexed cytometric bead array (CBA; BD Biosciences, CA, USA) run on an LSRFortessa Cell Analyzer (BD Biosciences, CA, USA). Data were acquired using DIVA Version 8.0 (BD Biosciences, CA, USA) and then analyzed using FCAP Array (Version 3; Soft Flow Hungary Ltd., Pécs, Hungary) to convert fluorescent intensity values into concentrations.

### 2.5. Statistical Analysis

Data are presented as mean ± standard deviation (SD) or median (quartile range) for data with a skewed distribution. Categorical data values are expressed as frequencies (percentages). Differences in their mean values between patients with candidemia or bacteremia and healthy controls were compared using an unpaired Student’s t-test and Mann–Whitney U test for skewed data distribution. Associations between two or more categorical variables (gender, patients and healthy controls with no apparent microbial infections) were examined using a chi-square (χ2) test or Fisher Exact test as appropriate. Key findings are presented using appropriate statistical graphs. All *p* values presented are two-sided and *p* values < 0.05 were considered as statistically significant. All statistical analyses were conducted using statistical packages SPSS 23.0 (SPSS Inc. Chicago, IL, USA) and Epi-info (Centers for Disease Control and Prevention, Atlanta, GA, USA) software.

## 3. Results

### 3.1. Descriptive Epidemiology

From January 2016 to December 2018, the sera of a total of 81 individuals were included in the study: 51 sera from patients with an episode of microbiologically proven candidemia, 20 sera from patients with bacteremia and 10 sera from healthy controls. Demographic and clinical data of candidemia patients are presented in Table 1. Two-thirds (66.7%) of the patients were male (n = 34), the mean age of candidemia patients was 41.09 ± 23.7. Risk factors for candidemia and hospital wards are shown in Table 1. The majority of candidemia patients 47/51 (92.2%) was at risk of malignancy, diabetics, surgery, neutropenia, central line and chemotherapy. For patients with candidemia, *Candida albicans* was the most common etiologic pathogen (n = 16, 31.4%), followed by *Candida glabrata* (n = 11, 21.5%), *Candida tropicalis* (n = 10, 19.6%), *Candida parapsilosis* (n = 7, 13.7%) and other yeast species (n = 5; 9.8%) (Table 1). For patients with bacteremia, Gram-negative bacteria were the most common pathogens (n = 17, 85.7%); these included eight *Escherecia coli*, two *Pseudomonas aeruginosa*, two *Klebsiella pneumoniae* and one isolate for each *Klebsiella oxytoca*, *Enterobacter cloacae*, *Acinetobacter baumannii*, *Citrobacter freundii* and *Moraxella catarrhalis*. Gram-positive bacteria were (n = 3; 15%) represented by two *Staphylococcus aureus* and one *Staphylococcus hominis*.

### 3.2. Interleukin Profile

**Figure 1:**
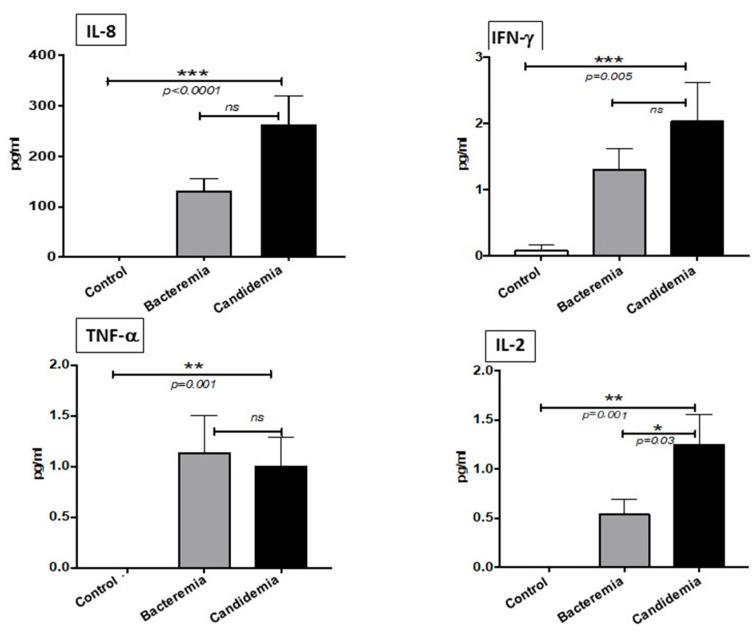
Concentration of Th-1 cytokines in serum. Serum was analyzed using multiplexed cytometric bead array. Histograms represent percentage change expressed as mean ± SEM (*p* > 0.05 is considered non-significant). The data represent triplicate measurements of interleukins. * = *p* < 0.05, ** = *p* < 0.01 and *** = *p* < 0.001.

We analyzed the Th-1 pro-inflammatory cytokines among the candidemia, bacteremia and control groups. Our results show that cytokines IL-8, IFN-γ, TNF-α and IL-2 were significantly elevated in the candidemia, compared to the control healthy group (Figure 1). This is in accordance with the notion that fungal infections induce the Th-1 cytokines in the serum of infected individuals. There was no significant difference between the candidemia and bacteremia group with respect to the cytokines IL-8, IFN-γ and TNF-α. Our results show that only IL-2 was significantly upregulated in the patients with fungal infection compared to the bacteria-infected patients.

**Figure 2:**
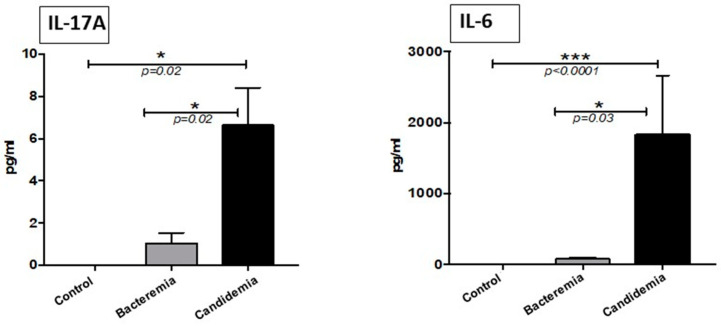
Concentration of Th-17 cytokines in serum. Serum was analyzed using a multiplexed cytometric bead array. Histograms represent percentage change expressed as mean ± SEM (*p* > 0.05 is considered non-significant). The data represent triplicate measurements of interleukins. * = *p* < 0.05 and *** = *p* < 0.001.

The most interesting result of our investigation was the significantly higher levels of the pro-inflammatory Th17 type cytokine, IL17A and IL-6, in the serum samples from patients with candidemia in comparison with samples from patients with bacterial infections and the healthy subjects (Figure 2). Serum IL-17A and IL-6 levels could not be detected in the healthy control group, whereas, in comparison with the bacterial group, the candidemia group had significantly elevated levels of these cytokines.

**Figure 3:**
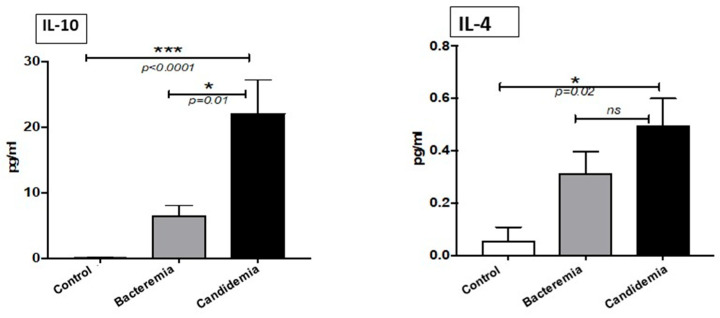
Concentration of Th-2 cytokines in serum. Serum was analyzed using a multiplexed cytometric bead array. Histograms represent percentage change expressed as mean ± SEM (*p* > 0.05 is considered non-significant). The data represent triplicate measurements of interleukins. * = *p* < 0.05 and *** = *p* < 0.001.

The levels of the anti-inflammatory cytokine IL-10 were significantly higher in the patients with candidemia than in both bacterial infected patients and normal healthy controls (Figure 3). Serum levels of IL-4 were significantly higher in the candidemia group than in healthy controls only. Even though there was an increase in the levels of IL-4 in the candidemia group compared to the bacteremia group, that was statically not significant (*p* = 0.009).

## 4. Discussion

We investigated whether or not the levels of Th-1, Th-2 and Th-17 cytokines would be useful for the diagnosis of candidemia and whether they existed in a different profile compared to bacteremia patient. Previous studies have found the levels of the inflammatory markers, C-reactive protein (CRP) and procalcitonin (PCT), to vary between bacteremia and candidemia groups [6,10,13]. Therefore, more potential biomarkers need to be discovered for differential diagnosis of bloodstream infections for patients in intensive care.

Th-1 cytokine IL-8, IFN-γ, TNF-α and IL-2, all of which are important pro-inflammatory cytokines and essential factors in innate immunity, were found in the present study significantly elevated in the patients with candidemia compared to normal healthy control subjects. Compared to bacteremia patients, candidemia patients had only IL-2 cytokine in significantly elevated levels. These results indicate that IL-2 cytokine may have a potential role in candidemia patients and can be exploited as a possible biomarker for the diagnosis after further evaluation. When infection occurs, it can lead to a systemic inflammatory response. However, other studies have shown conflicted findings in the serum level of IL-2, which were not significantly different in candidemia compared to bacteremia [10], whereas serum levels of IL-8, IFN-γ and TNF-α were non-significantly increased. Such findings were inconsistently reported in candidemia patients; while our results are in accordance with Atkin et al.’s [9], others reported a significant increase in the candidemia group compared to bacteremia patients [10]. Natural killer cells could regulate the IFN-γ function against fungal infection by directly killing the organism [14]. In addition, individuals with impaired IFN-γ signaling are at high risk of severe infection with *C. albicans* [15].

The present data showed IL-17A and IL-6 were significantly increased in candidemia patients compared to both bacteremia patients and normal healthy controls. These results are consistent with earlier published findings [6,7,10]. The IL-17A and IL-6 cytokines are specifically induced in the patients after *Candida* infection, making them a potential target for diagnostic purposes. The shielding role of Th-17 responses in the host defense against fungal infection was first established in IL-17A receptor-deficient (IL-17RA) mice, that showed increased susceptibility to systemic *C. albicans* infection [16]. In addition, deficiency in IL-17 led to a severe oropharyngeal candidiasis model in mice [17]. Several studies have shown IL-17A to play an important role in the development of the inflammatory response and host defense against *Candida* infections [9,10]. IL-17, now denoted as IL-17A, is the hallmark cytokine of the Th-17 cells and has been shown to function as a proinflammatory cytokine that upregulates a number of chemokines and matrix metalloproteases through the NF-_K_B and MAPK signaling pathways, leading to the recruitment of neutrophils into the sites of inflammation [18,19]. In an observational, prospective study, IL-17A levels were shown to be significantly increased in three patients with septic shock due to candidemia (primarily abdominal focus) compared to non-candidemic septic patients with or without *Candida* colonization, supporting the usefulness of IL-17A values for the diagnosis of invasive *Candida* infections [20]. In view of the limited ability to distinguish candidemia from bacteremia, IL-17A has to be considered as a biomarker for bloodstream infection rather than invasive *Candida* infections, as recently reported [21].

IL-6 is important to both innate and adaptive immunity [22]. It can be produced by many different cell types, including macrophages, endothelial cells and T cells. In addition to acting as part of the innate immune system, IL-6 induces C-reactive protein (CRP), fibrinogen and serum amyloid A to be expressed in hepatocytes [7]. As determined by clinical and laboratory characteristics, IL-6 concentrations correlate with the severity of sepsis [23]. IL-6-deficient mice are more susceptible to invasive candidiasis than wild type mice, which suggests that IL-6 release is fundamental during fungal infection [24].

In our study, IL-8 was not significantly increased in patients with *Candida* infections. IL-8 was reported to be an efficient predictor for bacteremia in most studies, while few publications have been concerned with changes in IL-8 levels during *Candida* infections [10].

Among the Th-2 cytokines, IL-10 and IL-4 were significantly elevated in candidemia patients compared to healthy controls, but only IL-10 was significantly elevated in candidemia compared to bacteremia groups. IL-10 is the cytokine released from macrophages and dendric cells (DCs) and the main function of this anti-inflammatory cytokine is to block the production of other cytokines from T helper-1 (Th-1) cells [25]. It was reported that IL-10 was reduced fivefold in renal transplant patients with invasive fungal diseases as compared to stable allograft recipients, which indicated that immunocompromised individuals could not respond to invasive fungal disease through IL-10 release [26]. It was found that patients with elevated IL-6 and IL-10 developed a higher proportion of health-care-associated infections, although this increase was not statistically significant [27].

Overall, our results indicate that a set of cytokines comprising IL-17A, IL-6, IL-2 and IL-10 is significantly elevated in the patient with bloodstream *Candida* infection as compared to the patients having bloodstream bacterial infections. Our study revealed that the cytokines IL-2, IL-6 and IL-10 were significantly higher in candidemia patients than in bacteremia patients, which was not reported in previous studies [9,10]. The levels were considered promising as a potential diagnostic marker. The study results could be exploited for differentiating and diagnosing *Candida* infections at an early stage by evaluating a larger cohort of patients. Early diagnosis of candidemia in high-risk patients using such high throughout testing may result in earlier medical treatment and improve the patient’s outcome.

## Figures and Tables

**Table 1:** Characteristics of the 51 critically ill candidemia patients and 20 patients with bacteremia.

Parameter	Candidemia	Bacteremia	
Age (y) mean ± SD	41.09 ± 23.7	43 ± 24.95	
Male (%)	34 (66.7)	11 (55)	
Mortality ≤ 30 days (%)	19 (37.3)	0.00	
Hospital ward. N (%)			
MICU	18 (35.3)	1 (5)	
SICU	9 (17.6)	1(5)	
PICU	7 (13.7)	0.00	
NICU	2 (3.9)	0.00	
TICU	0.00	3 (15)	
Oncology	5 (9.8)	4 (20)	
Surgery	6 (11.8)	5 (25)	
Non-ICU	4 (7.8)	9 (45)	
Malignancy N (%)			
Hematological diseases	8 (15.7)	1 (5)	
Solid tumor	8 (15.7)	3 (15)	
Medical/Surgical diagnosis N (%)			
DM	13 (25.5)	1 (5)	
History of Surgery	18 (35.3)	5 (25)	
Central line	15 (29.4)	0.00	
Tracheostomy/intubated	8 (15.70)	0.00	
Neutropenia	7 (13.7)	0.00	
Chemotherapy	10 (19.6)	3 (15)	
Nutrition (NGT/TPN)	6 (11.8)	0.00	
Dissemination	4 (7.8)	0.00	
Renal transplant	0.00	2 (10)	
Liver disease	0.00	1 (5)	
UTI	0.00	1 (5)	
None	0.00	3 (15)	
Species causing candidemia N (%)		Species causing Bacteremia	
*C. albicans*	16 (31.4)	*Escherichia coli*	8 (40)
*C. glabrata*	11(21.5)	*Pseudomonas aeruginosa*	2 (10)
*C. tropicalis*	10 (19.6)	*Klebsiella pneumoniae.*	2 (10)
*C. parapsilosis*	7 (13.7)	*Klebsiella oxytoca*	1 (5)
*C. dubliniensis*	2 (3.9)	*Enterobacter cloacae*	1 (5)
*Clavispora lusitaniae* (*C. lusitaniae*)	2 (3.9)	*Acinetobacter baumannii*	1 (5)
*Pichia kudriavzevii* (*C. krusei*)	1 (2.0)	*Citrobacter freundii.*	1 (5)
*Kluyveromyces marxianus* (*C. kefyr*)	1 (2.0)	*Moraxella catarrhalis.*	1 (5)
Non-*Candida* yeast	1 (2.0)	*Staphylococcus aureu.*	2 (10)
		*Staphylococcus hominis.*	1 (5)

## Data Availability

Not applicable.

## References

[1] Taj-Aldeen S.J., Salah H., Perez W.B., Almaslamani M., Motyl M., AbdulWahab A., Healey K., Perlin D.S. (2018). Molecular Analysis of Resistance and Detection of Non-Wild-Type Strains Using Etest Epidemiological Cutoff Values for Amphotericin B and Echinocandins for Bloodstream Candida Infections from a Tertiary Hospital in Qatar. Antimicrob. Agents Chemother..

[2] Wisplinghoff H., Bischoff T., Tallent S.M., Seifert H., Wenzel R.P., Edmond M.B. (2004). Nosocomial bloodstream infections in US hospitals: Analysis of 24,179 Cases from a prospective nationwide surveillance study. Clin. Infect. Dis..

[3] Playford E.G., Eggimann P., Calandra T. (2008). Antifungals in the ICU. Curr. Opin. Infect. Dis..

[4] Lamoth F., Lockhart S.R., Berkow E.L., Calandra T. (2018). Changes in the epidemiological landscape of invasive candidiasis. J. Antimicrob. Chemother..

[5] Lockhart S.R., Iqbal N., Cleveland A.A., Farley M.M., Harrison L.H., Bolden C.B., Baughman W., Stein B., Hollick R., Park B.J. (2012). Species identification and antifungal susceptibility testing of Candida bloodstream isolates from population-based surveillance studies in two U.S. cities from 2008 to 2011. J. Clin. Microbiol..

[6] Fu Y., Chen J., Cai B., Zhang J., Li L., Liu C., Kang Y., Wang L. (2012). The use of PCT, CRP, IL-6 and SAA in critically ill patients for an early distinction between candidemia and Gram positive/negative bacteremia. J. Infect..

[7] Tanaka T., Narazaki M., Kishimoto T. (2014). IL-6 in Inflammation, Immunity, and Disease. Cold Spring Harb. Perspect. Biol..

[8] Krause R., Zollner-Schwetz I., Salzer H.J.F., Valentin T., Rabensteiner J., Prüller F., Raggam R., Meinitzer A., Prattes J., Rinner B. (2014). Elevated Levels of Interleukin 17A and Kynurenine in Candidemic Patients, Compared with Levels in Noncandidemic Patients in the Intensive Care Unit and Those in Healthy Controls. J. Infect. Dis..

[9] Akin H., Akalin H., Budak F., Ener B., Ocakoğlu G., Gürcüoğlu E., Göral G., Oral H.B. (2015). Alterations of serum cytokine levels and their relation with inflammatory markers in candidemia. Med. Mycol..

[10] Wang Q., Wang C., Yang M., Li X., Cui J., Wang C. (2020). Diagnostic efficacy of serum cytokines and chemokines in patients with candidemia and bacteremia. Cytokine.

[11] De Pauw B., Walsh T.J., Donnelly J.P., Stevens D.A., Edwards J.E., Calandra T., Pappas P.G., Maertens J., Lortholary O., Kauffman C.A. (2008). Revised definitions of invasive fungal disease from the European Organization for Research and Treatment of Cancer/Invasive Fungal Infections Cooperative Group and the National Institute of Allergy and Infectious Diseases Mycoses Study Group (EORTC/MSG) Consensus Group. Clin. Infect. Dis..

[12] Abdul Wahab A., Taj-Aldeen S., Ibrahim E.B., Talaq E., Abu-Madi M., Fotedar R. (2015). Discrepancy in MALDI-TOF MS identification of uncommon Gram-negative bacteria from lower respiratory secretions in patients with cystic fibrosis. Infect. Drug Resist..

[13] Stoma I., Karpov I., Uss A., Krivenko S., Iskrov I., Milanovich N., Vlasenkova S., Lendina I., Belyavskaya K., Cherniak V. (2019). Combination of sepsis biomarkers may indicate an invasive fungal infection in haematological patients. Biomarkers.

[14] Morton C.O., Bouzani M., Loeffler J., Rogers T.R. (2012). Direct interaction studies between Aspergillus fumigatus and human immune cells; what have we learned about pathogenicity and host immunity?. Front. Microbiol..

[15] Chen G.-H., McDonald R.A., Wells J.C., Huffnagle G.B., Lukacs N.W., Toews G.B. (2005). The Gamma Interferon Receptor Is Required for the Protective Pulmonary Inflammatory Response to Cryptococcus neoformans. Infect. Immun..

[16] Huang W., Na L., Fidel P.L., Schwarzenberger P. (2004). Requirement of interleukin-17A for systemic anti-*Candida* albicans host defense in mice. J. Infect. Dis..

[17] Conti H.R., Shen F., Nayyar N., Stocum E., Sun J.N., Lindemann M.J., Ho A.W., Hai J.H., Yu J.J., Jung J.W. (2009). Th17 cells and IL-17 receptor signaling are essential for mucosal host defense against oral candidiasis. J. Exp. Med..

[18] Weaver C.T., Hatton R., Mangan P.R., Harrington L.E. (2007). IL-17 Family Cytokines and the Expanding Diversity of Effector T Cell Lineages. Annu. Rev. Immunol..

[19] Caron J.E., La Pine T.R., Augustine N.H., Martins T.B., Kumánovics A., Hill H.R. (2014). Severely depressed interleukin-17 production by human neonatal mononuclear cells. Pediatr. Res..

[20] Decker S.O., Sigl A., Grumaz C., Stevens P., Vainshtein Y., Zimmermann S., Weigand M.A., Hofer S., Sohn K., Brenner T. (2017). Immune-Response Patterns and Next Generation Sequencing Diagnostics for the Detection of Mycoses in Patients with Septic Shock—Results of a Combined Clinical and Experimental Investigation. Int. J. Mol. Sci..

[21] Wunsch S., Zurl C., Strohmaier H., Meinitzer A., Rabensteiner J., Posch W., Lass-Flörl C., Cornely O., Pregartner G., König E. (2021). Longitudinal Evaluation of Plasma Cytokine Levels in Patients with Invasive Candidiasis. J. Fungi.

[22] Scheller J., Chalaris A., Schmidt-Arras D., Rose-John S. (2011). The pro- and anti-inflammatory properties of the cytokine interleukin-6. Biochim. Biophys. Acta.

[23] Tsalik E., Jaggers L.B., Glickman S.W., Langley R.J., van Velkinburgh J., Park L.P., Fowler V.G., Cairns C.B., Kingsmore S., Woods C.W. (2012). Discriminative Value of Inflammatory Biomarkers for Suspected Sepsis. J. Emerg. Med..

[24] Romani L., Mencacci A., Cenci E., Spaccapelo R., Toniatti C., Puccetti P., Bistoni F., Poli V. (1996). Impaired neutrophil response and CD4+ T helper cell 1 development in interleukin 6-deficient mice infected with Candida albicans. J. Exp. Med..

[25] Redford P.S., Murray P.J., Ogarra A. (2011). The role of IL-10 in immune regulation during M. tuberculosis infection. Mucosal Immunol..

[26] Armstrong-James D., Teo I., Herbst S., Petrou M., Shiu K.Y., McLean A., Taube D., Dorling A., Shaunak S. (2012). Renal Allograft Recipients Fail to Increase Interferon-γ During Invasive Fungal Diseases. Arab. Archaeol. Epigr..

[27] Umberger R., Thompson C.L., Cashion A.K., Kuhl D., Wan J., Yates C.R., Muthiah M.P., Meduri G.U. (2015). Exaggerated plasma Interleukin 6, Interleukin 10, and Subsequent Development of Health Care–Associated Infections in Patients with Sepsis. Dimens. Crit. Care Nurs..

